# Coarse-Grained Clustering Dynamics of Heterogeneously Coupled Neurons

**DOI:** 10.1186/2190-8567-5-2

**Published:** 2015-01-12

**Authors:** Sung Joon Moon, Katherine A Cook, Karthikeyan Rajendran, Ioannis G Kevrekidis, Jaime Cisternas, Carlo R Laing

**Affiliations:** Department of Chemical and Biological Engineering & Program in Applied and Computational Mathematics, Princeton University, Princeton, NJ 08544 USA; School of Engineering and Applied Sciences, Universidad de los Andes, Av. Mons. Alvaro del Portillo, 12445 Santiago, Chile; Institute of Information and Mathematical Sciences, Massey University, Auckland, New Zealand

**Keywords:** Clustering dynamics, Heterogeneous coupling, Polynomial chaos expansion

## Abstract

The formation of oscillating phase clusters in a network of identical Hodgkin–Huxley neurons is studied, along with their dynamic behavior. The neurons are synaptically coupled in an all-to-all manner, yet the synaptic coupling characteristic time is heterogeneous across the connections. In a network of *N* neurons where this heterogeneity is characterized by a prescribed random variable, the oscillatory single-cluster state can transition—through  (possibly perturbed) period-doubling and subsequent bifurcations—to a variety of multiple-cluster states. The clustering dynamic behavior is computationally studied both at the detailed and the coarse-grained levels, and a numerical approach that can enable studying the coarse-grained dynamics in a network of arbitrarily large size is suggested. Among a number of cluster states formed, double clusters, composed of nearly equal sub-network sizes are seen to be stable; interestingly, the heterogeneity parameter in each of the double-cluster components tends to be consistent with the random variable over the entire network: Given a double-cluster state, permuting the dynamical variables of the neurons can lead to a combinatorially large number of different, yet similar “fine” states that appear practically identical at the coarse-grained level. For weak heterogeneity we find that correlations rapidly develop, within each cluster, between the neuron’s “identity” (its own value of the heterogeneity parameter) and its dynamical state. For single- and double-cluster states we demonstrate an effective coarse-graining approach that uses the Polynomial Chaos expansion to succinctly describe the dynamics by these quickly established “identity-state” correlations. This coarse-graining approach is utilized, within the equation-free framework, to perform efficient computations of the neuron ensemble dynamics.

## 1 Introduction

A network of oscillators can form sets of sub-networks oscillating with phase lags among them [1]; these are often referred to as *phase clusters*. The dynamic behavior of such states has been investigated experimentally in globally coupled photochemical oscillators [2–4]. Certain features of the cluster dynamics have been studied numerically and/or theoretically in a variety of contexts: in arrays of Josephson junctions [5], in networks of inhibitory reticular thalamic nucleus (RTN) neurons [6], in phase oscillators with nearest neighbor coupling [7, 8], in models for synthetic genetic networks [9], and for identical Hodgkin–Huxley (HH) neurons with homogeneous, weak coupling [10], to name a few.

In this paper we study a specific type of clustering dynamics observed in synaptically all-to-all coupled networks of identical HH neurons, but for which certain synaptic coupling parameters slightly vary across the population, thus making the whole network *heterogeneous*. The main feature underpinning the clustering dynamics is (approximate) symmetry. Aronson et al., taking a group-theoretic approach, study the bifurcation features in oscillator networks with  permutation symmetry, for an array of globally coupled homogeneous Josephson junctions [5]. Dynamical systems with such permutation symmetries are known to give rise to a large number of coexisting states—symmetrically related to one another—which is referred to as “attractor crowding” [11, 12]. Hansel et al. show that multiple Fourier mode interaction terms are necessary in a phase-reduced model of homogeneously all-to-all coupled identical HH neurons in order to account for multiple-cluster formation [10]. A generalization of the analytical framework for the Kuramoto-like coupled phase oscillators, including higher Fourier modes, has been attempted [13]. The dynamical nature of the transitions between different cluster states in phase-reduced oscillator models and slow switching along the heteroclinic orbits involved have been discussed [3, 14–19].

In these studies of cluster dynamics, it is often assumed that the constituting entities are identical and/or the coupling strength is weak, allowing dimensional reduction via complete synchronization within each cluster and/or through the phase reduction procedure. An actual population of neurons (or more generally, oscillators), however, would hardly be expected to satisfy this homogeneity assumption. In practice, *heterogeneity* often exists inevitably, and it can have consequences for the collective dynamics [20–23], which are not easily deduced from the dynamics in the homogeneous limit. In the presence of *weak* heterogeneity, it is natural to expect that similar oscillators (characterized by neighboring values of the heterogeneity parameter) may trace similar dynamical trajectories and tend to belong to the same sub-network, when the network splits into coherent sub-networks. However, the HH neuron networks we study here do not follow this intuitive expectation. The heterogeneity parameter value does not determine which sub-network the corresponding neuron would belong to; the heterogeneous parameter (which is drawn from an i.i.d. random variable) within *each* sub-network is statistically consistent with that of the *full* ensemble heterogeneity distribution.

We demonstrate a coarse-graining approach enabling the analysis of the low-dimensional dynamics of single- and double-cluster states, which provides an efficient way of studying the coarse-grained clustering dynamics of an arbitrarily large network. This work extends the approach introduced to study coarse-grained single cluster dynamics of networks of heterogeneous Kuramoto oscillators [24]. The approach is based on the *Polynomial Chaos (PC)*, also known as Wiener’s chaos expansion [25], originally introduced to model stochastic processes with Gaussian random variables using Hermite polynomials; it has been further developed and widely used for uncertainty quantification [26]. The PC-based approach utilizes the correlations that rapidly develop between the heterogeneity parameter values and the oscillator state variables. The same types of “identity-state” correlations are commonly observed to develop in a range of coupled oscillator models, including yeast glycolytic oscillators [27], van der Pol oscillators [28], and simplified neuron models [29].

The paper is organized as follows: The model and the parameter values used in it are described in Sect. 2, and some observations on the clustering dynamics in networks of heterogeneously coupled neurons are presented in Sect. 3. As a basis for understanding the dynamics of larger networks, the individual-level dynamics of a small number of neurons are analyzed in some detail (Sect. 4). A short survey of the PC expansion is provided and the dynamic behavior of large networks of neurons is studied in Sect. 5, while the derivation and the exploitation of our coarse-grained description of the clustering dynamics, utilizing the PC expansion, is presented in Sect. 6. The paper concludes with a brief summary and discussion.

## 2 Model

We study ensembles of Hodgkin–Huxley neurons. The dynamical state of each of the HH neurons is described by a set of variables , where *V* is the membrane potential, *m* and *h* are the activation and inactivation variables of the sodium current, and *n* is the activation variable of the potassium current. The equations for the *i* th neuron read [30]1

where *I* is the external current (an important control parameter in our study),  is the synaptic current entering the *i* th neuron (see Eq. (2) below), and 

for , and *n*, where 

We use almost the same parameter values as in [10], which correspond to a squid axon’s typical values at 6.3 °C: ; ; ; ; ; ; . The units for the parameters remain the same throughout the paper, and they are omitted hereafter, unless ambiguous.

The synaptic current for each neuron in a network of *N* all-to-all coupled neurons is modeled as 2

where , *g* is the coupling strength among the neurons (which is mostly set to 3.0 in this paper), and the synaptic variable  is governed by 3

The sigmoid  is chosen to be ; its exact functional form does, to some extent, affect the overall dynamics. The network of neurons we consider is heterogeneous in the following sense: each neuron has a different synaptic time constant  in Eq. (3). So even though the neurons are identical, they are coupled in a heterogeneous fashion, and there is one heterogeneous parameter () associated with each neuron. Assuming that , for *ω* we consider an i.i.d. uniform or normal random variable of zero mean value; however, the results presented below are not restricted to these particular choices of the heterogeneity distribution. Note that the normal distribution needs to be truncated so that  (hence ) is retained.

We choose the synaptic time *τ* to be heterogeneous, because overall clustering dynamics is sensitive to its variation. The presence of the heterogeneity in other parameters (such as *g*, , or *I*, as opposed to *τ*) would alter the detailed clustering dynamics in different ways (see Sect. 3 for details); however, whenever the strong correlation between the heterogeneity parameter and the variables develops in one way or another (see Sect. 3 for further details), the basic underpinning concept for the equation-free coarse-grained analysis presented in this paper can be again applied after an appropriate modification. In reality, all parameters are likely to be heterogeneous. The analysis of a network with multiple heterogeneities of this form is an interesting challenge, which is beyond the scope of the current study. In what follows, we consider the case , unless specified otherwise, and the width of the *ω* distribution (i.e., the standard deviation of *ω*, denoted by ) remains small compared to 1 so that the oscillators can still synchronize.

## 3 Cluster States

Given the parameter values presented in the previous section, an isolated neuron ( and ) undergoes a subcritical Andronov–Hopf bifurcation from a steady state as the external current reaches the value . The unstable periodic orbit born at this point eventually gains stability through a fold bifurcation of periodic orbits at . When *two neurons* are synaptically coupled together, the above-mentioned periodic orbits remain nearly unchanged, but the network exhibits bistability around the fold bifurcation point; the formation of clusters and the bistability itself has been well known [10]. Our study is focused on the parameter regime corresponding to this two-neuron bistability, but it addresses the case of many neurons, heterogeneously coupled together (which exhibit further multi-stability).

**Fig. 1:**
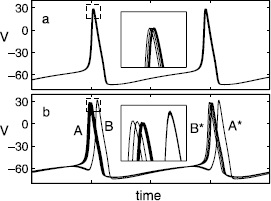
Time series of the membrane potentials (’s) of 10 heterogeneous neurons characterized by slightly different synaptic relaxation time constants  in Eq. (3); *ω* is uniformly distributed in  and . **a** Single-cluster state where the entire network oscillates synchronously (). **b** Double-cluster state (), whose period is approximately twice that of the orbit in panel **a**, where sub-networks of four and six neurons form two synchronously oscillating clusters, with a phase lag between them. *Insets* are blow-ups of the region at the peak of the action potential oscillations, marked by *dashed boxes*. In the double-cluster state in **b**, the cluster which “spikes first” on one cycle (denoted by *A*; consisting of six neurons) “spikes second” on the following cycle (A*), giving the solution its “period-2” nature (*B* and B* are the same group of four neurons at different time)

In this regime, the network can realize several types of stable periodic behavior: The entire network may oscillate synchronously (Fig. 1(a); single-cluster state), or may break up into two or more sub-networks (or clusters), each of them synchronously oscillating with a phase lag between the clusters (Fig. 1(b); double-cluster state). In each cluster, the trajectories of the constituting neurons get slightly “dispersed” as a consequence of the heterogeneity. The period of the double-cluster state is almost twice of that of the single cluster state (comparing Fig. 1(a) and (b)), and the transition between the single- and double-cluster states is related to the two-neuron period-doubling (PD) bifurcation (refer to Sect. 4 for further details). Such cluster states are observed for any network size.

**Fig. 2:**
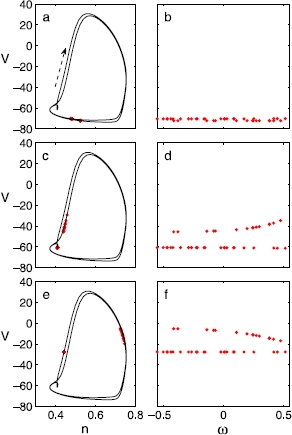
*Left panels*: Different snapshots of a double-cluster state of 40 neurons during a cycle, projected onto the *n*–*V* phase plane (), where *solid lines* are the limit cycles of a representative neuron; ’s are drawn from the uniform distribution. *The arrow* in **a** indicates the direction of the trajectories on the limit cycle. *Right panels*: *The dots* show the membrane potentials of all the neurons at the same time as in *the left panel*, plotted against their  values. In **d** and **f**, the naked eye can easily differentiate the two clusters. While the dynamic variables evolve, the correlations between the  and the  remain smooth throughout a cycle

The projection of a double-cluster state onto a certain phase plane of dynamical variables (right panels of Fig. 2) sheds light on an important aspect of the clustering dynamics; it reveals that (i) a strong correlation between the heterogeneity parameter *ω* and the dynamical variables *separately* develops for each cluster and (ii) that the neurons break up almost *evenly* in *ω* to form two clusters. The same type of correlation develops for the *entire* population of single-cluster states in a few different coupled oscillator models, which have been studied with a PC coarse-grained description and a subsequent equation-free analysis [24, 27–29]. Here, we attempt to extend this framework to apply to multiple-cluster states as well. Similar single- and double-cluster states exist also in homogeneous networks of identical HH neurons [10]; in the absence of heterogeneity, however, the constituting neurons of each cluster get synchronized *completely*, which naturally reduces the population-level dynamical dimension. In that case, each of the clusters can be treated as a fictitious neuron of appropriate weight or rescaling factor being assigned [31, 32], and the overall dynamics is effectively the same as that of a few/several neurons.

The splitting into two clusters can occur in a number of different ways (i.e., different permutations) at the individual neuron level, resulting in various distinguishable double-cluster states. For a given realization of *ω*, depending on the initial configuration, each cluster in a double-cluster state consists of different neurons; in other words, the value of  does not completely specify which cluster the *i* th neuron will join. Repeated simulations of various numbers of neurons with different initial configurations and/or independent random draws of *ω*, suggest that the sub-network sizes of the final stable double-cluster states tend to be almost the same ( neurons in one cluster and  in the other, where *ϵ* is a small integer satisfying ). We note that an apparently *different* type of, or an extreme permutation of, stable double-cluster state, where the neurons split at the “middle” value of , is still possible. This is indeed a legitimate permutation; however, such a state is—we believe—highly unlikely to occur spontaneously.

**Fig. 3:**
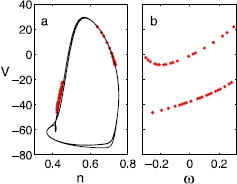
The same type of snapshot during a cycle as in Fig. 2, exhibiting a double-cluster state of 40 neurons, when the heterogeneity exists in a different parameter, the *coupling strength*: The coupling parameter is given as , where  and ’s are uniformly drawn from , while  and  throughout the neurons. As in the case of the heterogeneous time constant, the same type of bistability of the single- and double-cluster states is observed in the regime of the same parameter values considered here

**Fig. 4:**
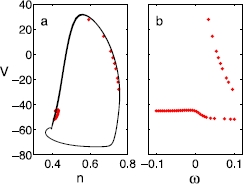
The same type of snapshot for 40 neurons as in Fig. 2, but when the heterogeneity exists in the *current*, , where  and ’s are uniformly drawn from . The other parameters that were heterogeneous in the previous figures, *g* and *τ* are kept at 3.0 and 1.0 throughout the neurons, respectively. In this case, a slightly different type of double-cluster states form, as well as the single cluster states. While the correlation still exists, only a specific portion of *ω* (of positive values) breaks up to form double clusters

We consider the cases where the heterogeneity exists in other model parameters as well. For instance, when *g* is given as , where  and *ω* is a uniform random variable, while *τ* is given as a fixed value of 1.0 throughout the neurons, we see almost the same behavior as in the case of a heterogeneous time constant presented above (Fig. 3). In case *I* is heterogeneous, in addition to the same type of single-cluster states, a slightly different type of double-cluster states are observed (Fig. 4); while the “identity-state” correlation still exists, only a specific range of *ω* breaks up to form double-cluster states. In this case, the coarse-graining method discussed in this study will have to be further extended; we will leave this as a topic for future study.

## 4 Background: Detailed Dynamics of a Few Neurons

The complete dynamical analysis of even a relatively small number of, say ten, neurons is highly complicated because the number of possible clustering states rapidly increases with  (scaling with the possible identity permutations). We obtain some initial insight into the generic dynamical features by analyzing small networks of neurons in detail.

**Fig. 5:**
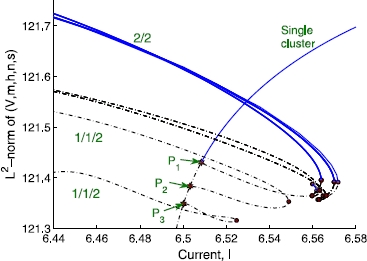
Bifurcation diagram for four heterogeneous neurons. Stable branches are represented by *solid (blue) lines*, while the unstable ones are *dotted (black) lines*. *Filled (red) stars* and *filled (red) circles* denote the period-doubling and fold bifurcation points, respectively

We start with four neurons, where ’s are distributed evenly in , i.e., ; at this population size, the characterization of the distribution function does not have statistical significance. The detailed bifurcation diagrams in this section are obtained using AUTO [33]. When the stable single-cluster (period-1) solution branch is continued in the decreasing direction of *I*, it loses the stability through a Period Doubling (PD) bifurcation, which is denoted by  in Fig. 5. As the current *I* decreases even further, there arise two more PD bifurcations ( and  in Fig. 5). Continuation of the bifurcated branch emanating from the first PD point  exhibits unstable period-2 double-cluster states, consisting of two clusters having two neurons in each cluster (referred to as a  split, where the notation  means *m* neurons belong to one cluster and the remaining *n* () neurons belong to the other). After a sequence of fold bifurcations (saddle-nodes of limit cycles, filled circles in Fig. 5), this unstable period-2 branch eventually becomes stable, giving rise to a stable double-cluster state of  split with a grouping of , i.e., neurons 1 and 4 form one cluster synchronously oscillating together, while neurons 2 and 3 form the other. For convenience, the neurons are labeled from 1 to *N* in the increasing order of the value for . Continuation of the solution branches bifurcated from  and  results in unstable three-cluster states with a  split, i.e., states for which two neurons oscillate in synchrony and the remaining two “clusters” contain one neuron each. These two branches also undergo numerous fold bifurcations, but they never gain stability.

**Fig. 6:**
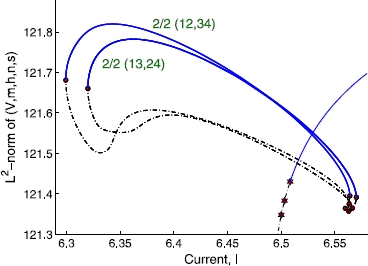
Bifurcation diagram for four heterogeneous neurons. Two isolas in the bifurcation diagram for the same four heterogeneous neurons as in Fig. 5. Each of the isolas corresponds to the splits of  and . Stable branches are represented by *solid (blue) lines*, while the unstable ones are represented by *dotted (black) lines*. *Filled (red) stars* and *filled (red) circles* denote the period-doubling and the fold bifurcation points, respectively

Starting from a stable  state, we obtain similar double-cluster states by swapping the dynamical variables among the neurons (while keeping ’s and all the other parameters unchanged) and directly integrating the model from this initial condition. We find that the solution branches for the other two combinations of evenly split double-cluster states of ,  and  states, are stable over a finite parameter range, forming *isolas* (Fig. 6; the single cluster state branch in Fig. 5 is included for comparison). The isolas have both stable (solid line) and unstable (dashed line) segments separated by fold bifurcation points (filled circles).

In the case of four neurons with *homogeneous* coupling (i.e., identical ), the above-mentioned three PD points ( through  in Fig. 5) collapse to a single point and the network exhibits a degenerate period-doubling bifurcation. Three Floquet multipliers simultaneously cross the unit circle at −1. All the cluster states of the same population size ratio then become indistinguishable, and for instance, the long term dynamics of single (double) cluster state is not different from that of a single (two) neuron(s).

**Fig. 7:**
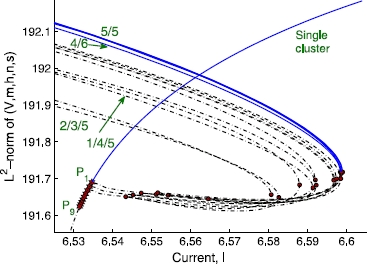
Bifurcation diagram for 10 heterogeneous neurons. Stable branches are represented by *solid (blue) lines*, while the unstable ones are represented by *dotted (black) lines*. *Filled (red) stars* and *filled (red) circles* denote the period-doubling and fold bifurcation points, respectively

The full bifurcation diagram for larger populations contains many more branches and bifurcation points, which rapidly becomes too complicated (and probably pointless) to analyze. It would help to consider the clustering dynamics of a few different progressively increasing network sizes, which would allow us to induce the dynamics of a larger network. Our analysis of four and ten neurons (see below) indicates that the essential features appear to remain the same. A bifurcation diagram for ten heterogeneous neurons including some of the relevant branches discussed above, is again obtained using AUTO. The ’s are again uniformly distributed in  for simplicity. The period-1 single-cluster state has nine PD bifurcations,  through  in Fig. 7; in general, there exist  PD bifurcations on the single-cluster solution branch of a network of *N* neurons. As in smaller networks, continuing the branch bifurcating from  leads to a double-cluster state with an equal-sized () split which eventually gains stability through a fold bifurcation. The continuation of branches bifurcating from  and  gives rise to unstable three-cluster states,  and , respectively, which never become stable. As was done with four neurons, we combinatorially swap the dynamical states of the neurons comprising a stable double-cluster, in order to obtain other stable double-cluster states. The original  split state has the grouping of  in the case of our random initial condition, where 0 represents the tenth neuron (). We obtained several other stable  splits, including , , , and even a stable  split, , all of which lie on solution branches forming isolas. These five stable branches are located very close to one another in phase and parameter space (Fig. 7). We expect that, in this version of “attractor crowding” [11, 12], small stochastic or deterministic perturbations may cause the dynamics to easily “flip” basins of attraction and approach nearby “coarsely indistinguishable” limit cycles. We checked other network sizes and found that, regardless of the population size, there exists a range of parameters for which multi-stability between single- and double-cluster states is observed, and the stable double-cluster states consist of nearly equal-sized clusters.

We now compare our results with the predictions by Aronson et al., regarding the equivariant system of a population with *homogeneous* coupling [5]. In the presence of  symmetry, the equivariant branching lemma [34] leads to  PD bifurcations on the single-cluster branch. The branches bifurcating off of the single cluster state branch are predicted to be of the form  (corresponding to  in our notation) with , where *p* and *q* are positive numbers representing the number of neurons in each cluster. Those authors showed that the double-cluster states may be stable for , and that the exact results for stability depend on the coefficients in the normal form at the bifurcations, which we do not attempt to obtain for the HH neuron model here. Other branches associated with other isotropy subgroups, the so-called “support solutions”, such as  with  for non-zero *p*, *q* and *r*, are predicted to emanate from the double-cluster state branches. Translating this prediction to the network of ten HH neurons, the  or  state is predicted to branch off from the single-cluster state, but  and  states may not directly branch off from there; rather, they may form as a consequence of subsequent bifurcations from the  or  double-cluster state. Our observations of the network with heterogeneous coupling are overall consistent with the above-mentioned predictions by Aronson et al. One non-trivial difference is that in our heterogeneously coupled HH neuron case, even three-cluster states with  and  groupings may branch directly off from the single-cluster branch (see Figs. 5 and 7).

## 5 A Coarse-Grained Description

### 5.1 Background: The Polynomial Chaos Expansion

In this subsection, we briefly review the polynomial chaos expansion method, which underpins the coarse-graining of the single- and double-cluster state dynamics in the following subsection. Wiener’s polynomial chaos expansion method [25], which has been widely used in the context of uncertainty quantification, allows one to obtain useful solutions to certain stochastic dynamical systems [26]. Consider a system described by a set of stochastic ODEs 4

where  is the *n*-dimensional model variable,  is the stochastic variable or parameter, an *m*-dimensional prescribed i.i.d. random variable each of which is drawn from the probability space . Here *Ω* is the sampling space, ℱ the *σ* field expanded by subsets of *Ω*, and *μ* the probability measure defined on ℱ. More complicated cases, e.g., where the dynamics is described by PDEs, can be formulated as well, however, such cases are not relevant to the current study.

Given a prescribed i.i.d. random variable *ω*, this method suggests the decomposition of the solution  in the Hilbert space of the appropriately chosen polynomials of the random variable: 5

where  is the member function or the basis function in the Hilbert space, and  is called the *i* th order PC coefficient. Here, a one-dimensional relation is considered for simplicity; however, the concept itself can be readily extended to cases of higher dimension for the functionals and/or the random variables. The basis polynomial functions are orthonormal in the sense that 6

where  is the complex conjugate of  and  is the Kronecker delta. From this orthonormality condition,  can be computed by 7

In practice, the above expansion gets truncated at a certain order. Previous studies [35, 36] confirm that the orthonormal polynomials chosen from the Askey scheme for a given probability measure ***μ*** make the PC expansion converge exponentially with the rate of , where *κ* is a constant. However, the number of PC coefficients may rapidly increase as the random variable dimension *m* becomes larger, posing a computational challenge.

For low-dimensional random dynamical systems, where faster convergence arises through the PC expansion, one can substitute the truncated expansion Eq. (7) into Eq. (4), 8

Taking the Galerkin projection on both sides using the basis , the following weak form [26, 36] is obtained: 9

consisting of a set of coupled ODEs for the PC coefficients , which provide an alternative description of the system dynamics to the original model, once such a description is confirmed to exist.

### 5.2 Coarse-Graining of the Clustering Dynamics

A computational dynamical analysis at the individual neuron level, such as the one presented in the previous section, is too complicated to perform for any realistic population size; a coarse-grained, population-level dynamical description and analysis become not only preferred, but necessary. Instead of keeping track of the state of every single neuron, we need to keep only a few collective descriptors of these states; yet, since the neurons are not homogeneous in their synaptic dynamics, a few moments of the distribution of the states are not sufficient: We need to not only know what the average and standard deviation of the states are, we also need to know *which neurons* (e.g. the low-*τ* or the high-*τ* ones) have low or high state values. In this joint distribution of neuron identities and neuron states, the *marginal* distribution of neuron states is not informative enough. That is why we turn to PC coefficients quantifying the correlation between the neuron *identities* and the neuron *states*. As was observed in the single cluster formation in a few different networks of oscillators [24, 27–29], a similar type of correlation between the dynamical variables (, , , ) of the *i* th oscillator and its heterogeneity parameter  rapidly develops in each of the clusters separately, during the initial transient (Fig. 2). The PC approach introduced to study the single cluster states [24] thus needs to be extended for the coarse-grained description of the double- and multiple-cluster states. In order to examine the possibility of applying the PC expansion to the double-cluster states, we first need to identify the distribution characteristics of the random (i.e., heterogeneity) parameters for *each* cluster, after the split.

When the network breaks up into two sub-networks, the original random parameters, ’s, are divided into two sets in a number of seemingly random ways, depending on the initial conditions of the neurons. Repeated numerical simulations from random initial configurations reveal that the random parameters for each cluster consistently span more or less the *same range* as the original random parameters (Fig. 2), and that the breaking of the original random parameter set into two subsets occurs in various permutations of the neuron identities. We quantitatively examine the statistical characteristics of the divided random parameters subsets using the Kolmogorov–Smirnov (KS) and the Wilk–Shapiro (WS) statistical tests [37], which compare the properties of an observed sample with those of the known distribution. As an illustrative example, we consider the case of a *normal* heterogeneity distribution.

**Fig. 8:**
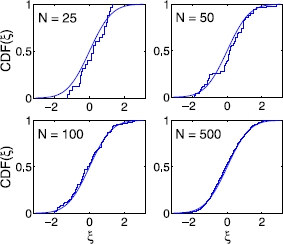
Kolmogorov–Smirnov test of the hypothesis that the heterogeneity distribution of one of the double-cluster states is consistent with the full heterogeneity distribution; this test is necessary in order to identify the characteristics of the random variable of each of the sub-networks, for possible PC expansion. The cumulative distribution function (CDF) of the normal distribution (*thin solid line*) is compared with the CDFs of  (*thick lines*) of sub-networks formed with 25, 50, 100, and 500 neurons, respectively. The original distribution for the entire population is a standard normal distribution

The KS test compares the quartiles, or the cumulative distribution functions (CDFs). Denoting the sample CDF and the CDF of a known distribution as  and *F*, respectively, let the largest difference between the two be 10

where *ω* is an i.i.d. random variable. For test statistics such as , the corresponding *p* value is the probability of obtaining a value of  at least as extreme as that observed. For a given *p* value, the threshold value of  can be computed. If  exceeds the threshold, then the distribution of the sample is said to be *inconsistent* with the assumed distribution with significance level *p*. For  below the threshold, all that can be said is that the distribution of the sample is *not inconsistent* with the assumed distribution characteristics, with significance level . The KS test is examined for the double-cluster states formed from a variety of initial configurations, in the case of the normal distribution of *ω*. When the population size exceeds hundreds of neurons, we find that the *p* value becomes very small, of the order of or even less than 0.01. The CDFs for varying network sizes are shown in Fig. 8. In addition to this, the WS test, comparing the ordered sample data with the expected value of the rank scores, or the normal scores or “rankits” [38], leads to the same conclusion.

**Fig. 9:**
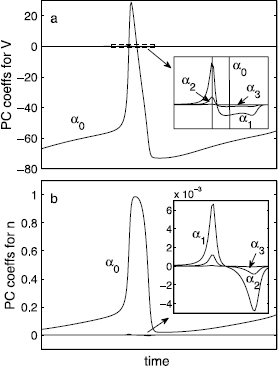
Time series of the first four PC coefficients (*α*’s) during a cycle for the variables of **a** *V* and **b** *n* (, ). A normal distribution of the standard deviation of 0.01 is used for *ω*, and the coefficients are expanded in Hermite polynomials. Only a single-cluster state case is shown, and the magnitudes of the corresponding coefficients in double-cluster states are comparable to single-cluster case. *The insets* are blow-ups of the region where the higher order coefficients have their maximum values; ’s in both cases are already practically negligible

Based on the above statistical tests, we conclude that the heterogeneity distribution *within each of the two sub-populations* is not inconsistent with the heterogeneity distribution of the entire population; and therefore, the same type of PC expansion used to coarse-grain the single-cluster state [24] can be applied to each of the double clusters *independently*, using the same basis functions and range. The PC expansion of the dynamical variables for each cluster reads 11

where  is the dynamical variable (e.g., *V*, *m*, *n*, or *h*) of the *i* th neuronal cluster, which is expanded up to the *l* th order in the basis polynomials .  is the *j* th order basis polynomial, which is chosen according to the characteristics of the random variable *ω*, following the generalized PC framework of the Askey scheme [36]. For instance, for a uniform random variable of *ω*, Legendre polynomials are the appropriate choice that leads to fast convergence. Likewise, Hermite polynomials [, , , , …] are appropriate for a normal random variable (Fig. 9) as in Wiener’s original work [25]. In the end, the states of 100 neurons in two clusters can be summarized in terms of a few PC coefficients per state variable per cluster; in our case 100 neurons (400 total variables, excluding synaptic variable) will be seen to usefully reduce to three coefficients per state variable (and thus 12 variables) for each cluster, for a total of 24 variables, a reduction by a factor of 16.7; 200 or even 2000 total neurons would still reduce to 24 coarse variables!

In an infinitely large network where the distribution of the random variable can be treated as continuous, the coefficients  can be determined by the orthogonality relationship among the basis functions (Eq. (7)). However, in a finite-size network, as is often the case in practice, or when a truncated distribution is considered (e.g., the normal distribution for the current system, with the constraint of ), the orthogonality no longer holds exactly, and regression, such as a least squares fitting, determines the PC coefficients better. The *j* th PC coefficient for a particular variable *y* at a given time *t* is obtained by minimizing the residual  of the *i* th cluster 12

where  is a variable associated with the *k* th neuron belonging to the *i* th cluster, which consists of  neurons. The first two coefficients have the following geometrical meaning on the coarse-grained level:  is the average value, and  measures the level of linear spread of the variable among the neurons around the average value , as a consequence of the heterogeneity. For the case of the membrane potential (when  is ),  measures the average potential, and  roughly measures the instantaneous spread of the potential among the neurons in the *i* th cluster. The higher order PC coefficients are related to higher order moments of the spread of the individual neuron’s variables in each cluster.

The individual-level details, such as the exact composition of the neurons in each cluster, vary among different initial conditions and different draws of the random variable *ω*. However, the temporal trajectories of the PC coefficients remain robust over such microscopically distinguishable states, with a small level of statistical fluctuation. The PC expansion Eq. (11) converges rapidly; the magnitudes of  rapidly decrease with increasing *j* (Fig. 9), as expected from the Askey scheme. Upon ensemble averaging, the PC description provides an appropriate statistical representation of the coarse-grained state.

So far, the random parameters in the divided clusters are assumed to be described by the same distribution as the original one for the entire network based on the findings of the statistical tests. However, even if statistically unlikely, the previously mentioned extreme case of “split-in-the-middle” state where one cluster is formed by the neurons of  ( is a specific value around the middle value of 0) while the other cluster consists of neurons with the remaining values of *ω*, does exist; an artificially prepared double cluster state conforming to this grouping (whether  or not) is indeed found to be stable. There exist only few limit cycle solutions of this type, and such states would be statistically insignificant in the coarse-grained description. Should such a split arise, the heterogeneity characteristics of each sub-network is clearly *inconsistent* with the full heterogeneity distribution. In this case, the heterogeneity sub-domain corresponding to each cluster should be treated separately to account for the split at . A variant or extension of the multi-element PC method developed for stochastic differential equations [39] should be considered in this case.

## 6 Coarse-Grained Computations

In this section, we perform equation-free coarse-grained computations for double cluster states, treating each of them separately. By doing this, we circumvent the steps deriving the model equations for the PC coefficients (Eq. (9)) for the current system. We do not identify the coarse-grained model equations; however, we analyze the dynamics by computationally obtaining the solutions to those equations. This approach does not rely on any simplifying assumptions, such as weak coupling, as long as synchronization occurs. It is not limited to a particular choice of the distribution for the random parameters and, in principle, it works equally well both for “large” finite and infinite network sizes. The success of this method attests to the accurate and sufficient description of the network by the chosen few coarse-grained variables.

In order for a coarse-grained calculation of double-clusters to be feasible, the neurons belonging to different clusters need to be systematically identified and grouped together. This can be done in the following way: As the variation of the dynamical variables within a cluster is much less than that between two clusters most of the time during a cycle (Fig. 2), the neurons belonging to different clusters can be differentiated by measuring the temporal correlation of their dynamical variables. The time series of the neuron variables observed over a length of time  (still a fraction of the period) is sampled at a set of intervals, say at every time interval of ; then the correlation of the sampled time series is calculated. A threshold is applied to the correlation matrix; matrix entries are set to 1 if above the threshold and 0 otherwise. The thresholded correlation matrix can be interpreted as the adjacency matrix of the network of neurons. The first non-trivial eigenvector of the adjacency matrix reveals clustering of the neurons. The entries of this eigenvector are clearly clustered around two distinct values. Projections of the eigenvector onto the different neurons are sorted by these values, thereby identifying two clusters.

We start the equation-free coarse-grained analysis by integrating the double-cluster states in time, using a forward Euler coarse projective integration method [40] (which does not differ conceptually from the coarse integration of single-cluster states). The first *three* PC coefficients for each dynamical variable are retained as the coarse-grained variables (truncating at  is enough for general purposes, per the convergence results seen in Fig. 9). This method, in which a forward Euler—or other choice—projection algorithm is directly applied to the time evolution of the coarse-grained variables, is the simplest demonstration of the applicability of equation-free computations. Each iteration in this algorithm consists of a few steps; healing, microscopic evolution (direct integration of the full model), and the projection of the coarse-grained variables. The number of time steps in each of the healing, direct integration, and jump steps can be fixed for the entire time evolution or be adaptively changed for better efficiency [41].

**Fig. 10:**
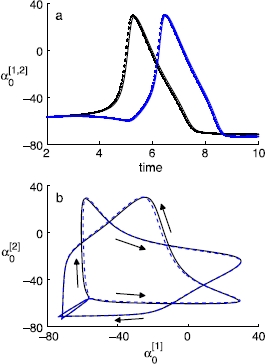
Coarse projective integration of double-cluster states (*dotted lines in blue and black colors*), compared against the direct full integration (*solid lines in the same colors*). Both projective integrations are performed with a fixed step size of 0.001; after five healing steps, three such steps during a short direct integration are used to estimate time derivatives, with a forward Euler jump of 20 steps. **a** The blow-up of the temporal trajectory of  for *V* (the average membrane potential) of two clusters, during a fraction of the period, where the two clusters reach the peak potential successively. **b** The  for *V* of one cluster against that of the other cluster, during a cycle of a double-cluster state (, with the standard deviation  is 0.05). *Arrows* indicate the direction of the evolution over time

We implement the coarse projective integration algorithm with fixed time-step sizes for each of the healing, evaluation, and jump steps, selected to accommodate accuracy and stability even when the variables rapidly change around a spike; these step sizes are almost certainly “overly cautious” during the slow recovery phase of the neuron. The small errors between coarse projective integration and direct full integration (which depend on the projection step size and the projection method) can be seen in Fig. 10. Projective integration for the cluster states needs to be implemented with caution because of the network’s multi-stability.

**Fig. 11:**
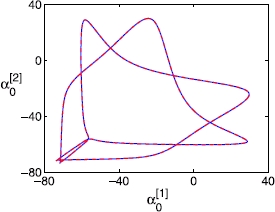
Limit cycles of a double-cluster state calculated with a coarse-grained fixed point algorithm for 100 neurons, plotted in terms of the first order PC coefficient  (for *V*) of one cluster against that of the other cluster (). A normal distribution of *ω* is considered, with a standard deviation of 0.01 and . *The solid line (blue)* corresponds to the full integration, and *the dashed line (red)*, which overlays *the solid* one, corresponds to what is obtained by the coarse fixed point algorithm. Note that these two solutions are visually indistinguishable

A more sophisticated algorithm can be used to compute the coarse-grained periodic solution of the double clusters, utilizing an equation-free fixed point algorithm [40] and the PC expansion. Standard Newton–Raphson-like fixed point algorithms require evaluation of both the coarse residual and the coarse Jacobian. The coarse-grained level equations are evaluated with the coarse time-stepper, and the evaluation of the coarse Jacobian is circumvented using a Krylov subspace method, which requires only evaluating *the action of this Jacobian* on specified vectors [42]. The coarse-grained representation of a typical solution is found this way. The individual and coarse solutions can deviate slightly, as only a finite number of PC coefficients are used during the computations, but the difference is practically unnoticeable in the “eye norm” (Fig. 11).

Both of the demonstrated equation-free algorithms successfully compute the correct dynamical states, confirming that a few PC expansion coefficients are appropriate coarse-grained dynamical variables, enabling the description and the dynamical analysis of the large ensemble of neurons at that level.

## 7 Conclusion

Any “system of systems” in practice, including a network of neurons studied here, is unlikely to consist of homogeneously coupled identical entities, and the consideration of heterogeneity among the constituent entities is often necessary. The heterogeneity may introduce fundamental differences into the dynamics, compared to the homogeneous case. The oscillating entities in each cluster are now no longer completely synchronized, the dynamical dimension of the network increases tremendously, and the individual-level dynamics and the corresponding dynamical analysis in a traditional way could be much more complicated. Even though some of the qualitative features may remain the same as in the homogeneous case, the detailed dynamics often cannot easily be deduced from that of the homogeneous limit. Furthermore, the system size is often finite, and an analysis treating it as an infinitely large system, where the heterogeneity parameter is assumed to be continuous, might not be appropriate. The equation-free coarse-grained computational method presented here is well suited to such cases, without requiring any of simplifying assumptions.

In a heterogeneously coupled network of Hodgkin–Huxley neurons, as studied here, all the combinatorially different ways of transitioning between the single- and the double-cluster states are distinguishable at the individual neuron level. The composition of the neurons in each of the double-cluster states is apparently “randomly” decided, depending on the initial configuration. We see that the random variables in each sub-network are statistically *not inconsistent with* those of the original, entire network. This enables each of the double clusters to be described and analyzed in the equation-free polynomial chaos framework that has already successfully been applied to single-cluster states in several other examples. Our approach, based on the strong correlations among the variables which rapidly develop in each of the clusters separately during the initial transient, gives rise to a low-dimensional description of a large heterogeneous network. The approach is applicable to a range of oscillator models exhibiting the same type of splitting of random parameters in the formation of double clusters. Though this work focuses on neurons splitting into two groups (in networks of Hodgkin–Huxley neurons), the techniques used here constitute a first step that can be extended for different types of oscillators and different number of groups or clusters, as long as the correlation remains valid. For instance, in the case of slightly different types of double clusters (when *I* is heterogeneous; refer to Fig. 4), our method should be extended, in line with some variant of the multi-element PC method developed for stochastic differential equations [39].
